# Factors Affecting Pheromone Production by the Pepper Weevil, *Anthonomus eugenii* Cano (Coleoptera: Curculionidae) and Collection Efficiency

**DOI:** 10.3390/insects5040909

**Published:** 2014-11-18

**Authors:** Fred J. Eller, Debra E. Palmquist

**Affiliations:** 1 Functional Foods Research Unit, National Center for Agricultural Utilization Research, USDA-ARS, 1815 North University Street, Peoria, IL 61604, USA; 2 Midwest Area Office, Agricultural Research Service, United States Department of Agriculture, 1815 North University Street, Peoria, IL 61604, USA; E-Mail: Deb.Palmquist@ARS.USDA.gov

**Keywords:** *Anthonomus eugenii*, *Capsicum annum*, aggregation pheromone, volatile collection, porous polymer adsorbent, male age, diel periodicity, male density, diet

## Abstract

Several factors affecting pheromone production by male pepper weevils, *Anthonomus eugenii* Cano (Coleoptera: Curculionidae) as well as collection efficiency were investigated. Factors studied included: porous polymer adsorbents (Tenax *versus* Super Q), male age, time of day, male density, and male diet. Super Q was found to be a superior adsorbent for the male-produced alcohols and geranic acid as well as the plant-produced E-β-ocimene. Pheromone production increased with male age up to about age 15 days old and then tapered off. Male pepper weevils produced the highest amount of pheromone between noon and 2 pm (*i.e.*, 4 to 6 h after “lights on”) and were producing *ca.* 800 ng/h during this period. Thereafter, pheromone production decreased and was extremely low during the scotophase (*i.e.*, *ca.* 12 ng/h). Male pepper weevil density had a significant effect on both release rate and pheromone composition. Pheromone production on a per male basis was highest for individual males and the percentage of geranic acid in the blend was lowest for individual males. Male pepper weevils produced only extremely low amounts of pheromone when feeding on artificial diet; however, they produced very high amounts when on fresh peppers. Together, this information will be useful in designing better attractant lures for pepper weevils.

## 1. Introduction

The pepper weevil, *Anthonomus eugenii* Cano (Coleoptera: Curculionidae), is an important pest of both sweet and hot peppers (*Capsicum* spp.) in the southern United States and throughout Central America [1,2]. The most important damage is yield reduction resulting from premature abscission of infested fruit. Infested fruit not aborted may contain frass and decaying plant tissue making them unmarketable. Additionally, the pepper weevil has been implicated in the transmission of internal mold of peppers [3]. Because the pepper weevil larvae and pupae are protected within the environment of the pepper pod, insecticide treatments must be directed against the emerged adults. Effective chemical control of adult pepper weevils requires detecting adults prior to economic injury [4]. A predictive model for pepper weevil adult emergence is unavailable; however, both a damage-based threshold [5] and a visual count method using adult pepper weevils on terminal buds have been described [6,7]. Action thresholds for the pepper weevil are low: 5% terminal bud damage [5] and between 1 adult/400 terminals [7] and 1 adult/100 terminals [6]. Because both sampling methods are tedious, time-consuming and may only detect weevils after they have passed economic levels, a better monitoring system is needed for this pest.

Male pepper weevils have been demonstrated to produce an aggregation pheromone attractive to both male and female pepper weevils in the field [8]. The pheromone blend was identified and found to contain six male-specific compounds: (*Z*)-2-(3,3-dimethylcyclohexylidene) ethanol, (*E*)-2-(3,3-dimethylcyclohexylidene) ethanol, (*Z*)-(3,3-dimethylcyclohexylidene) acetaldehyde, (*E*)-(3,3-dimethylcyclohexylidene) acetaldehyde, (*E*)-3,7-dimethyl-2,6-octadienoic acid (geranic acid), and (*E*)-3,7-dimethyl-2,6-octadien-1-ol (geraniol) [9]. A commercial trap based on this attractant is available (Trece Inc., Adair, OK, USA) and has been demonstrated to be a sensitive method to detect pepper weevils even in low population levels [10]. They also reported a positive linear relation between trap catch and fruit damage.

During the isolation and identification of the aggregation pheromone of the pepper weevil it was necessary to maximize the collection of the pheromone to perform the chemical analyzes. The information obtained could also be useful for improving the attractiveness of synthetic lures used to manage of pepper weevils as well. The objectives of this study were to investigate several factors which could affect pheromone production by male pepper weevils or pheromone collection in an effort to optimize pheromone collection for identification purposes and to investigate potential effects of several environmental factors. The factors studied included: (1) a comparison of porous polymer adsorbents; (2) effect of male age; (3) effect of time of day; (4) effect of male density; and (5) effect of male diet. The null hypothesis was that none of these factors would have an effect on the total amount of pheromone or the ratio of pheromone components collected.

## 2. Experimental Section

### 2.1. Insects

A reproducing laboratory colony of pepper weevils was established from insects collected in Florida. Pepper weevils were reared according to methods described [8]. Fresh jalapeño peppers were purchased locally, grown in a greenhouse, or grown in an outdoor garden. Emerging adult pepper weevils were held individually in 30-mL diet cups and fed sliced fresh jalapeño pepper, or a piece of artificial diet [11]. Adult pepper weevils were sexed using CO_2_ anesthetization and males were identified by the presence of a large hooked metatibial spur (mucro) [12].

### 2.2. Collection of Volatiles

Early in our research on the male pepper weevil pheromone [9], we investigated the possible use of solvent extraction to accumulate sufficient material for pheromone identification. Several male pepper weevils which had been used in a volatile collector and were known to be producing pheromone (*i.e.*, a mean of over 20 μg/male day) were soaked overnight in *n*-hexane and the solvent subsequently analyzed by gas chromatography (GC). The GC analyses indicated that the extract contained an average of less than 500 ng/male (*i.e.*, *ca.* 2.5% of the amount collected by volatile collection). Therefore, it was clear that volatile collections would provide much higher amounts of material as well as avoiding potential interferences co-extracted by the solvent. Subsequently, only volatile collections were used to collect materials.

Volatile collections were made using the volatile collection system previously described [9]. Briefly, the system consisted of a 20-cm × 2.2-cm ID Pyrex glass tube sealed on each end with a #11 cork stopper. One cork held a prefilter (7-cm × 4-mm ID glass tube) with *ca.* 6 mm of porous polymer (*ca.* 23 mg) held between a stainless steel screen (325 mesh; F. P. Smith Wire Cloth Co., Franklin Park, IL, USA) and a glass wool plug. The other cork held a similar filter with *ca.* 4 mm of porous polymer to collect volatiles. Air was drawn through the tube with either the house vacuum system or a vacuum pump at a flow of *ca.* 130 mL/min.

Collected volatiles were extracted from the filters using 240-μL hexane for Tenax filters and methylene chloride for Super Q filters. Ten microliters of a 250 ng/μL solution (*i.e.*, 2500 ng) of α-terpineol was added to each filter extract as an internal standard to quantify collected volatiles and calculate pheromone release rates.

### 2.3. Gas Chromatography

Gas chromatography was performed using a Hewlett-Packard 5890 Series II gas chromatograph (GC) equipped with a flame ionization detector with helium as the carrier gas and a Spectra-Physics SP4400 integrator. The column used was a fused silica Hewlett-Packard HP-5 (0.17-μm film thickness, 25 m × 0.32 mm ID) (Hewlett Packard Co., Avondale, PA, USA). The linear flow velocity was 28 cm/s. The temperature program was: 50 °C for 3 min then 10 °C/min to 220 °C. The injector and detector temperatures were 170 °C and 250 °C. Injections of 1–2 μL were made in the splitless mode and changed to the split mode after 0.60 min.

### 2.4. GC-Mass Spectrometry (GC-MS)

Electron-impact mass spectra (El-MS) were obtained on a Hewlett-Packard 5970 Mass Selective Detector. An ionizing potential of 70 eV was used for EI spectra. Sample introduction was through a Hewlett-Packard 5890 GC fitted with a DB-1 (0.25 μm film thickness, 15 m × 0.25 mm ID) capillary column using the same program described for gas chromatography.

### 2.5. Standard Volatile Collection Conditions

Pepper weevils were typically held individually and fed small jalapeño peppers. Pepper weevils were held under a 14:10 (L:D) photoperiod and the temperature was held constant at 27 °C.

### 2.6. Porous Polymer Adsorbents

Two porous polymers adsorbents were compared: Tenax (60–80 mesh, Alltech Associates, Inc., Deerfield, IL, USA) [13] and Super Q porous polymer (80/100 mesh; Alltech Associates, Inc., Deerfield, IL, USA) [14]. The two adsorbents were tested in tandem pairs; Tenax before Super Q and Super Q before Tenax. Equal amounts of each were used as described above under Collection of Volatiles. Our analyses of the collected volatiles of pepper weevils also revealed the release of several plant-derived compounds including a very high amount of E-β-ocimene which was typically the most abundant compound collected. This compound was identified by mass spectral analyses and confirmed by comparison to a commercial sample (International Flavors and Fragrances Inc., Hazlet, NJ, USA). This compound was among several plant-derived reported to be released by peppers damaged by pepper weevils [15]. The collection efficiency of the porous polymers towards this compound was also examined.

### 2.7. Effect of Male Age

Volatiles were collected daily from male weevils from age 1 to 30 days old. The extraction of the filters from the volatile collections were made at “lights on”. Volatiles were collected using Super Q filters from three replications of male pepper weevils at each age.

### 2.8. Effect of Time of Day 

There were six Time of Day collections made per day. The lights came on 8 am. The first collection was made at 2 h after “lights on” (*i.e.*, collection from 8 am–10 am), and subsequent collections were made at 4 (*i.e.*, collection from 10 am–noon), 6 (*i.e.*, collection from noon–2 pm), 9 (*i.e.*, collection from 2 pm–5 pm), 14 (*i.e.*, collection from 5 pm–10 pm) h after lights on. The collection at 14 h after lights on was made at “lights off”. The last collection was made at lights on at 8 am (*i.e.*, collection from 10 pm–8 am). Volatiles were collected using Super Q filters from 5 individual male weevils over 5 days beginning at age 4 days old and ending at age 8 days old. The extracts for the five individual males were pooled for a given time of day and age for a total of 30 GC analyses.

### 2.9. Effect of Male Density

Three densities of male pepper weevils were tested: 1, 2 and 5 weevils per volatile collector. Volatiles were collected using Super Q filters from 3 replicates of male weevils for 5 consecutive days beginning at age 3 days old and ending at age 7 days old.

### 2.10. Effect of Male Diet

Preliminary attempts to collect pheromone from males feeding on artificial diet failed to produce significant amounts of pheromone even after several weeks. Therefore, the effect of male diet on pheromone production was investigated by starting male pepper weevils on one diet and switching the diet after four days. One group began on small jalapeño peppers and was switched to artificial diet, while the other group started on artificial diet and was switched to small jalapeño peppers. Volatiles were collected using Super Q filters from 3 replicates of male weevils over 10 days beginning at age 1 day old and ending at age 10 days old.

### 2.11. Statistical Analyses

Independence of the time point data was tested using a Durbin-Watson statistic from a best-fit weighted regression analysis of Ln (total pheromone) as a function of time squared. The weighting used was 1/variance. Levene’s test for homogeneity of variance was conducted prior to analysis of variance (ANOVA) to assure statistical assumptions were met. Data for total pheromone was transformed by natural log transformation to stabilize the variance and to match the data used for Durbin-Watson testing. Means were compared by least significant difference (LSD) with Bonferroni’s adjustment. Statistical analyses were performed using SAS [16], and TableCurve 2D software [17].

## 3. Results and Discussion

### 3.1. Porous Polymer Adsorbents

The results of the comparison of porous polymer adsorbents are shown in Table 1. The ANOVA indicated there was a significant effect of adsorbent on alcohols collected (*F*_3,8_ = 39.2; *p* = 0.0000). When the volatiles passed first through the Tenax filter and then the Super Q filter, only a very low percentage (*i.e.*, *ca.* 7%) of the total male-produced alcohols were trapped on the Tenax filter. Conversely, when the volatiles passed first through the Super Q filter, virtually all (*i.e.*, 99.8%) of the male-produced alcohols were trapped on the Super Q filter. The ANOVA indicated there was a significant effect of adsorbent on geranic acid collected as well (*F*_3,8_ = 29.8; *p* = 0.0001). Tenax was more effective at capturing the male-produced geranic acid than the alcohols and Tenax retained *ca.* 92% of the total geranic acid collected. However, Super Q was even more effective and it retained 98% of the total geranic acid collected when the Super Q was the first filter. Although the pepper weevil pheromone blend includes two aldehydes, they are only present in relatively minor amounts and were not analyzed separately.

**Table 1:** Comparison of effectiveness of porous polymers for collecting volatiles ^a^. Super Q is represented by SQ.

Compound	Tandem Pair		Tandem Pair	
	Tenax before SQ SQ after Tenax		SQ before Tenax Tenax after SQ	
	Mean (SEM) Amount Collected (ng)			
Male-Produced Alcohols	1227 (48) b	15869 (2778) a	19034 (1447) a	38 (23) b
Male-Produced Acid	1594 (168) a	140 (5) b	1674 (279) a	34 (34) b
Plant-Produced Ocimene	4873 (2043) b	16166 (1635) a	19312 (3078) a	396 (198) b
	Mean (*n* = 3) Percent of Tandem Pair Total			
Male-Produced Alcohols	7.2	92.8	99.8	0.2
Male-Produced Acid	91.8	8.1	98.0	2.0
Plant-Produced Ocimene	23.2	76.8	98.0	2.0

^a^ Means (*n* = 3) for a tandem pair without letters in common differ significantly using LSD (*p* = 0.05).

The ANOVA indicated there was a significant effect of adsorbent on ocimene collected (*F*_3,8_ = 19.8; *p* = 0.0005). The Super Q filter before Tenax captured the highest amount of ocimene, however, it was statistically equivalent to the Super Q filter after Tenax. The efficiency of Tenax for capturing E-β-ocimene was intermediate between the male-produced alcohols and male-produced geranic acid, with *ca.* 23% of the total E-β-ocimene captured on Tenax when it was the first in the tandem pair. Again, Super Q was even more effective and it retained 98% of the total E-β-ocimene collected when the Super Q was the first filter.

### 3.2. Effect of Male Age

**Figure 1:**
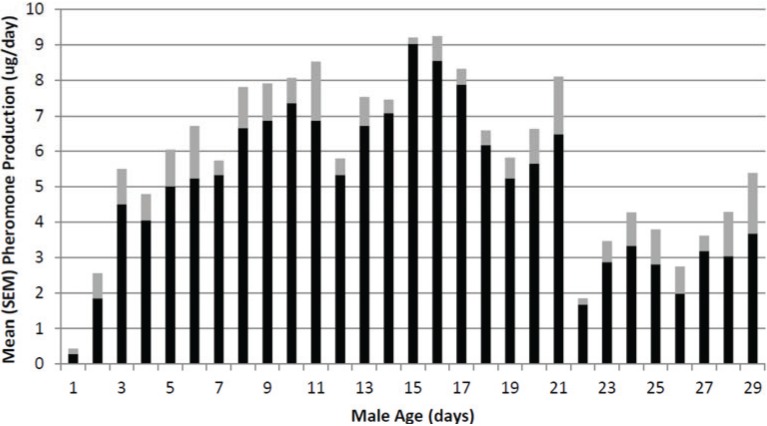
Effect of male pepper weevil age on pheromone production. Values represent means (black bar) and standard deviation of the mean (SEM) (gray bar).

The effects of male age on total pheromone production are shown in Figure 1. Pheromone production started out very low (*i.e.*, less than 1 μg/day) and then increased to a maximum at age 15 days old of *ca.* 9 μg/day. Afterward, pheromone production decreased as the male pepper weevils increased in age. However, even at age 30 days old, the male pepper weevils were producing over 3 μg/day and these weevils continued to produce pheromone an additional 7 days until the experiment was finally ended.

### 3.3. Effect of Time of Day

**Figure 2:**
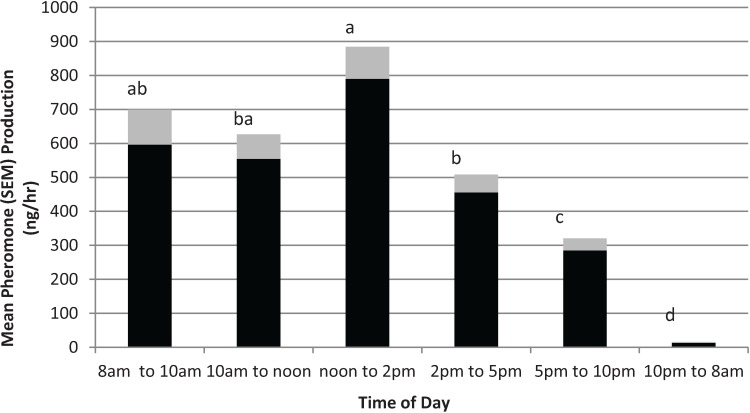
Effect of time of day on male pepper weevil pheromone production. “Lights on” at 8am and “lights off” at 10 pm. Values represent means (solid bars) and standard deviation of the mean (SEM) (gray bar) and are means over 5 days of collections. Means without letters in common differ significantly using LSD (*p* = 0.05).

The effects of time of day on male pheromone production are shown in Figure 2. The Durbin-Watson test revealed a first order autocorrelation of only 0.145, indicating effectively no dependence between the time point data. The *F*-value for Levene’s test for homogeneity of variance was 0.87 (*p* = 0.51) demonstrating that the natural log transformed data met the statistical assumptions for ANOVA. The ANOVA indicated there was a significant effect of time of day on total pheromone collected (*F*_5,20_ = 43.4; *p* = 0.0000). During the scotophase (*i.e.*, 10 pm to 8 am), male pepper weevils produced very little pheromone (*i.e.*, *ca.* 12 ng/h). However, as soon as the lights came on (*i.e.*, the photophase), pheromone production increased dramatically to *ca.* 600 ng/h. The period between noon and 2 pm gave the highest pheromone release rate of *ca.* 800 ng/h. After 2 pm, pheromone production gradually tapered off to between 300 and 400 ng/h.

### 3.4. Effect of Male Density

**Figure 3:**
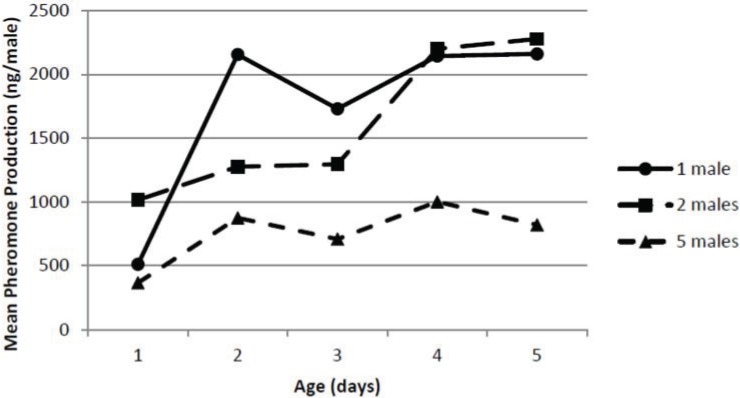
Effect of male density and age on pheromone production.

The effect of male pepper weevil density on total pheromone production is shown in Figure 3. The ANOVA indicated there were significant effects of both male density (*F*_2,30_ = 7.39; *p* = 0.0025) and age (*F*_4,30_ = 3.39; *p* = 0.021) on total pheromone collected but not a significant interaction between density and age (*F*_8,30_ = 0.62; *p* = 0.754). The individually held pepper weevils showed a similar increase in age to that seen in the male age study although at a slightly lower level. When five male pepper weevils were held together, the total pheromone release rate on a per male basis did not increase much with age and was generally about half (*i.e.*, *ca.* 1 μg/male day) of what was observed for the individual male pepper weevils. The total pheromone release rate on a per male basis for the two weevils held together was overall similar to that seen for the individual male pepper weevils, and was, in fact, statistically equivalent.

**Figure 4:**
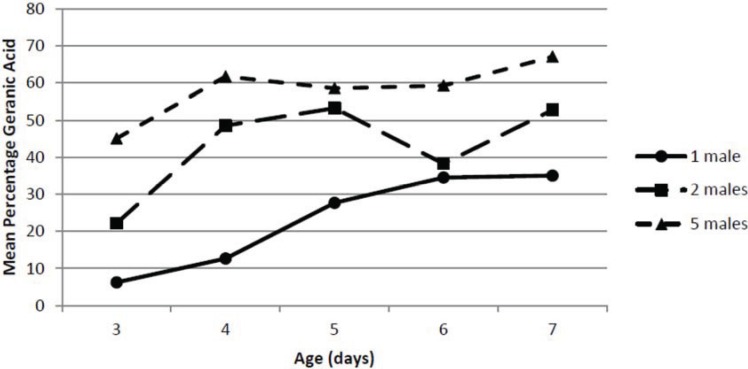
Effect of male density and age on percentage geranic acid.

Changes in the percentage of geranic acid in the pheromone blend were extremely obvious, while the relative percentages of the other components were fairly consistent. The effect of male pepper weevil density on the percentage geranic acid in the total pheromone blend is shown in Figure 4. Again, the ANOVA indicated there were significant effects of both male density (*F*_2,30_ = 51.6; *p* = 0.0000) and age (*F*_4,30_ = 10.57; *p* = 0.0000) on percentage geranic acid but not a significant interaction between density and age (*F*_8,30_ = 1.54; *p* = 0.186). For all densities of male pepper weevils, the percentage geranic acid tended to increase slightly with male age. However, there was a very strong effect of male density on the percentage geranic acid and the percentage geranic acid was proportional to male pepper weevil density. At a density of 5 males at age 7 days old, the percentage geranic acid in the total blend was *ca.* 67%, whereas for individually held pepper weevils it never exceeded *ca.* 36%. The percentage geranic acid produced by the density of two male pepper weevils was intermediate between that of 1 and 5 male pepper weevils. Although it is possible that at the density of 5 males there could have been breakthrough of the pheromone which would have led to a lower total pheromone release rate on a per male basis, the fact that the geranic acid increased with density suggests that that is not the case.

### 3.5. Effect of Male Diet

**Figure 5:**
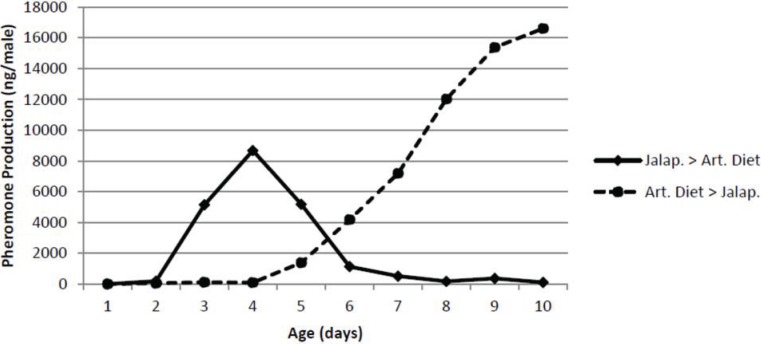
Effect of switching diet on pheromone production. The two treatments were jalapeno followed by artificial diet (Jalap. > Art. Diet) and artificial diet followed by jalapeno (Art. Diet > Jalap.).

The effect of male diet on pheromone production by male pepper weevils is shown in Figure 5. As expected, the male weevils started on the small jalapeño peppers increased pheromone production with age and were producing *ca.* 8 μg/male day at the point when they were switched to artificial diet. Thereafter, their pheromone production immediately decreased and after 3 days on the artificial diet, they produced only extremely low amounts of pheromone. On the other hand, the male pepper weevils on the artificial diet for up to four days produced essentially no pheromone even though identically aged males on jalapeño peppers were producing substantial amounts of pheromone. Once these males were switched to jalapeño peppers, they began producing pheromone immediately and after only 4 days, they were producing over 10 μg/male day.

## 4. Conclusions

The superiority of the Super Q polymer adsorbent was clearly evident for the three compounds examined in this study. These compounds represent three distinct types of chemical functionality, namely, alcohol, carboxylic acid and hydrocarbon. The fact that Super Q captured a higher percentage of each suggests it would be a better adsorbent for a wide range of compounds.

In addition to the amount of individual components retained, the observed percentages of the alcohols and geranic acid in the mixture are extremely different depending on which filter was first. For example, when the Tenax filter was before the Super Q filter, the percentage geranic acid in the mixture was over 50%. However, when the Super Q filter was before the Tenax filter, the percentage geranic acid in the mixture was only *ca.* 8%. This lower value is undoubtedly closer to what the male pepper weevils are actually releasing. The type of filter used could give very different estimates of the pheromone composition.

Pheromone production by males is affected by male age and, in the lab, seemed to be highest for weevils aged *ca.* 2 weeks old. The highest release rate measured for the weevils in the age study was *ca.* 9 μg/day. The time day of day study indicated that was a definite diel periodicity to pheromone release. The highest release rate was between noon and 2 pm (*i.e.*, 4 to 6 h after “lights on”) and was *ca.* 800 ng/h. Because a synthetic formulation must release pheromone over a 24 h period rather than just during the photophase, a synthetic formulation to be used for field trapping should have an hourly release rate close to the highest hourly rate observed (*i.e.*, *ca.* 19 μg/day) rather than the average daily rate (*i.e.*, 9 μg/day).

Although it may seem reasonable to attempt to collect pheromone from a large number of insects to obtain the most pheromone possible for chemical studies, the effect of density should not be ignored since the density can have a significant effect on both release rates as well as pheromonal composition. Pepper weevils did not make as much pheromone on a per weevil basis when held in groups. This effect, if not considered, could have a large impact on the calculated pheromone release rate. In addition, and perhaps more importantly, the density of the male pepper weevils had a very significant effect on the composition of the collected pheromone. The percentage of geranic acid was nearly doubled when five males were held together. The biological significance of this difference is unknown. It is possible that a group of male pepper weevils producing a higher percentage of geranic acid may be more attractive than an individual pepper weevil. Conversely, the group could be less attractive. In any event, the effect of various ratios of geranic acid in the pepper weevil pheromone blend may yield important information to optimize the pheromone blend’s attractiveness. If the presence of the geranic acid led to a decreased attractiveness of the blend, blanketing an area containing pepper weevils with geranic acid could lead to a decrease in mating success of pepper weevils seeking mates and aid in managing this pest.

It would have been more convenient to study the pheromone of the pepper weevil if it could have easily done without the need for fresh peppers and the work entailed in maintaining live pepper plants. However, to identify the pheromone using weevils on the artificial diet used in this would have been immensely more difficult. It may be possible to add something to the artificial diet to improve it to the point where the pepper weevils would produce more pheromone. Because the artificial diet did not contain any components derived from peppers, an extract of peepers would seem a logical starting point to improve the diet for pheromone production. It may also be that some physical characteristic of the peppers is at least partly responsible as well.

Additionally, the of use fresh peppers allowed for the collection of plant-derived compounds from the weevil-plant complex. The release of these plant-derived compounds may have a role in combating the insect itself or pathogens. It is also likely that these compounds could have an effect on the attractiveness of the weevil-plant complex. The pepper weevil’s long-range attraction to plant volatiles has been described [18] and the attractiveness of volatiles from damaged peppers to pepper weevils and the chemical identity of several compounds, including ocimene has been reported [15]. The addition of these compounds to the pheromone blend could have a significant effect on its attractiveness and improve the effectiveness of lure traps as has been demonstrated for boll weevils [19].
